# Emerging diagnostic and therapeutic strategies for urothelial carcinoma

**DOI:** 10.1080/2090598X.2021.1876203

**Published:** 2021-01-14

**Authors:** Marco Moschini, Rafael Sanchez-Salas

**Affiliations:** Department of Urology, Luzerner Kantonsspital, Luzern, Switzerland, marco.moschini87@gmail.com; Department of Urology, Institut Mutualiste Montsouris and Université Paris Descartes, Paris, France

Urothelial carcinomas (UCs) represent the fourth most frequent tumours in developed countries [1]; among these, bladder cancer (BCa) is the 10th most common form of cancer worldwide, with 549 000 new cases and 200 000 deaths estimated in 2018 [2], while upper urinary tract UC (UTUC) accounts for 5–10% of UCs [1]. Recently, the European Association of Urology (EAU) and the European Society of Medical Oncology (ESMO) selected a panel of experts to define ‘hot topics’ in the BCa field, in order to improve current management and to propose new solutions [3,4]. In >50 years of BCa care a limited amount of improvement has been verified in terms of cancer-specific survival and quality of life for patients harbouring the disease. Just recently, comprehensive understanding of BCa biology has changed the status of tumour treatment and today we have not only improved our approach to localised disease, but we are providing effective treatment for more advance stages. In this regard, it is important to continue the efforts in Urological Oncology to improve survival and quality of life of patients with BCa even in these difficult times with the entirety of humanity challenged by the well-known coronavirus disease 2019 (COVID-19) pandemic.

Therefore, we are grateful to the *Arab Journal of Urology* and to the Editor-in-Chief, Professor Shokeir, for giving us this unique opportunity and challenge to keep on supporting thoughtful BCa research.

In this Special Issue on BCa and UTUC, we collected a series of articles treating some of the most important and innovative topics on the subject. First, the use of biomarkers in the form of inflammation markers or questionnaires (such as the Frailty Score) to individuate prognostic outcomes and to select the optimal treatment in patients with BCa, even in the postoperative management. Second, new imaging techniques are being implemented in this field to improve sub-stratification and offer the optimal treatment to our patients. In this issue, we will evaluate the role of multiparametric MRI and fluorodeoxyglucose-positron emission tomography (FDG-PET) as emerging tools in the diagnosis of BCa. Third, novel treatments have been recently developed in the field of non-muscle-invasive BCa, especially the role of programmed death-ligand 1 (PD-L1) and the effect of electro-motive drug administration (EMDA) in a subgroup of patients. Fourth, we also give some space to UTUC, reporting about optimal surgical technique and the need for lymph node dissection.

The readers will find interesting clinical information that can be readily taken to the patient’s side and ideally included into prospective trial assessment. The UC path as we know today could elegantly be challenged with novel medical tools and information with the objective of improving patient’s outcomes.

We would like to thank once again the editorial team, the Editor-in-Chief, authors, reviewers and all the readers for their efforts in putting together robust work to further improve research and care of patients with UC.
